# Identification of S100A9 as a Potential Inflammation-Related Biomarker for Radiation-Induced Lung Injury

**DOI:** 10.3390/jcm12030733

**Published:** 2023-01-17

**Authors:** Youyi Liu, Mengdi Wu, Jingrou Guo, Yifei Tang, Hongliang Jiang, Bo Yang, Minchen Wu, Jianfeng Huang

**Affiliations:** 1 Wuxi School of Medicine, Jiangnan University, Wuxi 214122, China; 2 Department of Radiation Oncology, Affiliated Hospital of Jiangnan University, Wuxi 214122, China

**Keywords:** radiation-induced lung injury, S100A9, radiotherapy, inflammation, biomarkers

## Abstract

Radiation-induced lung injury (RILI), a potentially fatal and dose-limiting complication of radiotherapy for thoracic tumors, is divided into early reversible pneumonitis and irreversible advanced-stage fibrosis. Early detection and intervention contribute to improving clinical outcomes of patients. However, there is still a lack of reliable biomarkers for early prediction and clinical diagnosis of RILI. Given the central role of inflammation in the initiation and progression of RILI, we explored specific inflammation-related biomarkers during the development of RILI in this study. Two expression profiles from the Gene Expression Omnibus (GEO) database were downloaded, in which 75 differentially expressed genes (DEGs) were screened out. Combining Gene Oncology (GO), Kyoto Encyclopedia of Genes and Genomes (KEGG) analysis and protein–protein interaction (PPI) network analysis, we identified four inflammation-related hub genes in the progression of RILI—MMP9, IL-1β, CCR1 and S100A9. The expression levels of the hub genes were verified in RILI mouse models, with S100A9 showing the highest level of overexpression. The level of S100A9 in bronchoalveolar lavage fluid (BALF) and the expression of S100A9 in lung tissues were positively correlated with the degree of inflammation in RILI. The results above indicate that S100A9 is a potential biomarker for the early prediction and diagnosis of the development of RILI.

## 1. Introduction

Radiotherapy is an important mainstay for the treatment of multiple thoracic malignancies, such as lung cancer and esophageal cancer [1]. However, the susceptibility of the lung to radiation contributes to the occurrence of radiation-induced lung injury (RILI), among the most common and serious complications during thoracic radiation and limits the efficacy of radiotherapy [2,3]. The incidence rate of RILI is up to 15% among patients treated with thoracic radiotherapy, despite constant technological advances in conformal radiotherapy [4]. Clinically, RILI remains a great obstacle that negatively affects the local control rates of thoracic cancers and the long-term quality of life in patients [5]. RILI is typically divided into early reversible pneumonitis, occurring within hours to a few days after irradiation exposure, and irreversible advanced-stage fibrosis, occurring months to years after the radiotherapy [6,7]. Thus, early identification of patients at high risk for RILI for the purpose of instituting early preventive measures is crucial to improving prognosis.

Studies have revealed that inflammation plays a central role in the development of RILI [8]. Several minutes after irradiation, the generation and accumulation of reactive oxygen species (ROS) and nitrogen species (NGS) lead to DNA damage and cell death [9]. Inflammatory cells subsequently infiltrate the affected region to remove dead cells and release cytokines and chemokines, including tumor necrosis factor-α (TNF-α), interleukin-1β (IL-1β), interleukin-6 (IL-6), and transforming growth factor-β1 (TGF-β1) [10]. All the inflammatory factors above further amplify the inflammatory effect through a series of reactions and cause the persistence of the inflammatory state, culminating in early reversible toxicity (pneumonitis) and even contributing to irreversible late toxicity (fibrosis) [11]. Thus, it is of great importance to explore the specific inflammation-related biomarkers and their underlying mechanisms during RILI progression for the development of novel diagnostic and therapeutic strategies in RILI.

In this study, two mRNA microarray datasets of RILI mouse models downloaded from the Gene Expression Omnibus (GEO) were analyzed and the DEGs between RILI tissues and normal controls shared in both of the datasets were screened out. Then, inflammation-related biomarkers and their underlying mechanisms in RILI were investigated via Gene Ontology (GO) enrichment, Kyoto Encyclopedia of Genes and Genomes (KEGG) pathway enrichment and protein–protein interaction (PPI) network analysis. In the end, the hub genes identified were S100A9, MMP9, IL-1β and CCR1 and they were verified via qRT-PCR, ELISA and immunofluorescence staining in RILI mouse models. The results showed that S100 calcium-binding protein A9 (S100A9) could be a potential prediction and diagnosis target of development of RILI.

## 2. Materials and Methods

### 2.1. Microarray Data Acquisition and DEGs Identification

GSE25295 [12] and GSE41789 [13] profiles were screened from the Gene Expression Omnibus (GEO) (http://www.ncbi.nlm.nih.gov/geo/, assessed on 10 January 2022), a publicly available database of gene/microarray profiles. GSE25295 is based on the GPL1261 (Affymetrix Mouse Genome 430 2.0 Array) platform and contains 3 lung tissues from mice with 25 Gy radiation and 4 normal controls harvested in the sixth week after irradiation. GSE41789 is based on the GPL1261 (Affymetrix Mouse Genome 430 2.0 Array) platform and includes 3 tissue samples from mice with 17.5 Gy radiation and 3 normal controls harvested in the eighth week after irradiation.

The DEGs between RILI samples and normal samples of the two datasets were identified using the online tool GEO2R (https://www.ncbi.nlm.nih.gov/geo/geo2r/, assessed on 10 January 2022). The values for statistical significance were set as the adjusted *p* value < 0.05 and |logFC| > 1.0. The shared DEGs of the two datasets were identified using the Venn diagram webtool (http://bioinformatics.psb.ugent.be/webtools/Venn/, assessed on 1 February 2022).

### 2.2. GO and Pathway Enrichment Analyses

GO analysis is used to explore the biological processes of high-throughput transcriptome or genome data, covering three distinct aspects of gene function—biological process (BP), cellular component (CC) and molecular function (MF) [14]. The genes enriched in inflammation-related biological processes in GO analysis were screened out and composed the “inflammation term”. KEGG, a collection of databases that integrates genomic, chemical, biological and systemic functional information, provides a link between genes and higher-order processes, such as pathways [15]. In this work, the Database for Annotation, Visualization and Integrated Discovery (DAVID) (https://da-vid.ncifcrf.gov/, assessed on 1 February 2022) was used to visualize the shared DEG enrichment of the BP, MF, CC and pathways (false discovery rate < 0.05).

### 2.3. PPI Network Creation and Hub Gene Identification

The shared DEGs enriched in the inflammation term were listed as the primary objective and submitted to the Search Tool for the Retrieval of Interacting Genes (STRING) (http://string-db.org/, assessed on 1 February 2022), an online database used for analyzing the functional protein association networks. Afterward, PPI pairs with a combined score > 0.40 were extracted and the PPI relationship network was constructed by Cytoscape software (version 3.6.1). Finally, a plug-in CytoHubba of Cytoscape was utilized to filter the hub genes by calculating the degree of each protein node, then ranked the genes according to the degrees. In this study, 4 genes were identified as hub genes.

### 2.4. Animals

C57BL/6J male mice (6- to 8-wk-old) were acquired from the Changzhou Kavins Experimental Animal Co. Ltd. (Changzhou, China). The mice were housed (12 h light/dark cycle) under pathogen-free conditions at 24 ± 2.0 °C and 55 ± 10% humidity and allowed free access to water and standard rodent chow food (four mice/cage). All animal experiments were carried out according to the guidelines of the Institutional Animal Care and approved by the Animal Ehtics Committee of Jiangnan University (JN. No20211130c0800630[508]). 

### 2.5. Lung Irradiation Protocol

A total of 36 mice were randomly and equally distributed into two groups: an irradiation group and a control (non-irradiated) group. The mice in the irradiation group received a single fraction thoracic irradiation. After anesthesia with isoflurane, the mice in the irradiation group were fastened on a custom lead mold and received 25 Gy of X-ray irradiation at a dose rate of 3 Gy per minute using a linear accelerator (Elekta Synergy, Elekta Limited, Crawley, England). When mice were irradiated, a 1.0 cm-thick bolus was used to correct the distribution of radiation. Irradiation characteristics were beam energy, 6-MV photons; source–surface distance, 100 cm; size of the radiation field, 30 × 2 cm^2^; gantry, 180°. Dosimetry was measured with a cylindrical ionization chamber before irradiation. While the mice in the control group received the same treatment as the irradiation group except for irradiation.

### 2.6. Sampling

Mice were euthanized at 2, 3 and 4 weeks after irradiation along with controls. Bronchoalveolar lavage fluid (BALF) was harvested by cannulation of the trachea. A syringe with 0.5 mL PBS was inserted thrice along with the lung bronchus for tracheal lavage. After being centrifuged at 1000 r for 5 min at 4 °C, the supernatant was obtained and stored at −80 °C. Lungs were collected and divided into two pieces: one fixed in 4% paraformaldehyde for histological analysis, and one stored at −80 °C waiting for RNA extraction.

### 2.7. Histopathology and Immunofluorescence

After soaking in 4% paraformaldehyde for 48 h, the lung tissue was fixed in 10% buffered formalin solution, embedded in paraffin wax, sectioned and subsequently stained with hematoxylin and eosin (H&E) according to routine protocols. Briefly, after deparaffinization and rehydration, tissue sections were stained with hematoxylin solution (Solarbio, Beijing, China) for 5 min followed by 5 dips in 1% acid ethanol (1% HCl in 75% ethanol) and then rinsed in distilled water. Afterwards, the sections were stained with eosin solution (Solarbio, Jiangsu, China) for 3 min and followed by dehydration with graded alcohol and clearing in xylene. Three mounted slides were randomly selected from each group and examined using a LEICA DM3000 LED (Leica DMshare (v3), Wetzlar, Germany). Five different fields of view per slice were evaluated for the infiltration of inflammatory cells. Based on previous reports, inflammation scores were determined by measuring the infiltrated inflammatory cells in the alveolar and interstitial space [16].

Immunofluorescence staining was performed to assess the expression of S100A9 and neutrophils (Ly6g+) in lung tissues of the RILI mouse model. The lung tissues soaking in 4% paraformaldehyde were embedded in paraffin and sliced. Sections of lung tissues were dewaxed, rehydrated and antigen repaired. After being blocked with 10% goat serum, the sections were subsequently incubated with rabbit anti-mouse S100A9 (26992-1-AP, Proteintech, Wuhan, China, dilution: 1:200) at Ly6g (65140-1-Ig, Proteintech, Wuhan, China, dilution: 1:200) 4 °C overnight, and incubated with Cy3 goat anti-rabbit lgG (SA00009-2, Proteintech, Wuhan, China, dilution: 1:50). After incubation with anti-fluorescence quencher (containing DAPI) at room temperature for 5 min, the stained lung slides were evaluated under a Zeiss Axio Imager Z2 fluorescence microscope (Carl Zeiss Micro imaging GmbH, Jena, Germany). The quantification of fluorescence intensity was analyzed using Image J software (version 1.8.0).

### 2.8. Real-Time Quantitative RT-PCR and ELISA

The total RNAs of mice lung were extracted with Total RNA Extraction Reagent (Yeasen Biotechnology, Shanghai, China) from the lung tissues stored at −80 °C and cDNA synthesis was performed with a One-Step RT-PCR Kit (CoWin Biosciences, Taizhou, Jiangsu, China). The mRNA expression levels of the 4 hub genes (GAPDH as the internal control gene) were detected by SYBR Green-based real-time PCR (qPCR SYBR Green Master Mix, Yeasen Biotechnology, Shanghai, China and LightCycler 480 II, Roche, Basel, Switzerland). All results of the 4 hub genes were taken relative to quantification based on the 2-ΔΔCt algorithm, and all primers of targets and internal control genes are itemized in Table 1.

BALF in mice was utilized to measure the expression level of S100A9 in lung. ELISA was performed with commercial ELISA kits (Meimian, Nanjing, Jiangsu, China) according to the manufacturer’s instructions.

### 2.9. Statistical Analysis

Each trial of each sample had three technical replicates. Data were expressed using the mean and the standard deviation. An independent-samples t test was performed to identify significant differences between the control and treatment groups using SPSS 17.0 software. A *p* value of less than 0.05 was considered statistically significant.

## 3. Results

### 3.1. Determination of DEGs in RILI

**Figure 1:**
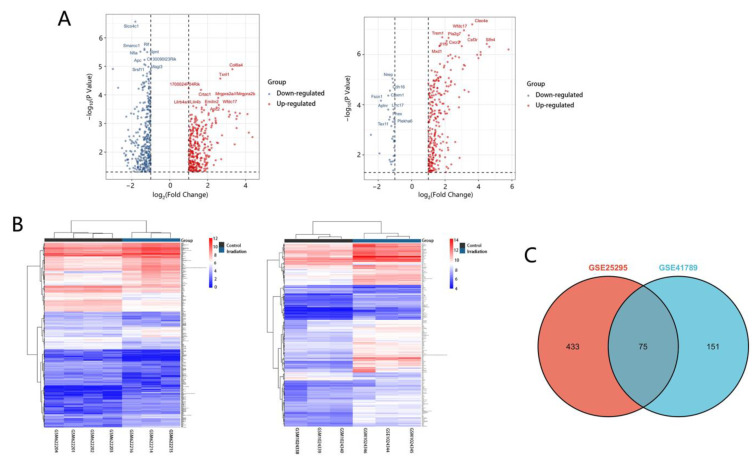
Volcano plots, heatmap and Venn of DEGs. (**A**) Volcano plot analysis identifies DEGs. Red dots represent up-regulated genes and green dots represent down-regulated genes. The differences are set an |log FC| > 1. (**B**) Heatmap of DEGs identified in two datasets. Red areas represent highly expressed genes and green areas represent lowly expressed genes. (**C**) Venn diagram of common DEGs from the two datasets. There are 75 common DEGs in the two datasets.

To identify DEGs in RILI, we downloaded relevant expression profiles from two microarray datasets (GSE25295 and GSE41789). In total, 647 and 297 DEGs were extracted from GSE25295 and GSE41789 based on the defined criteria. The DEGs are shown in the volcano plots and the heatmaps (Figure 1A,B), of which GSE25295 included 308 up-regulated genes and 339 down-regulated, and GSE41789 included 266 up-regulated genes and 31 down-regulated genes. The shared DEGs of the two datasets were integrated using the Venn Diagram online tool, including 73 up-regulated and 2 down-regulated genes, among which 47 genes were homologous to the human counterparts. (Figure 1C, and Supplementary Table S1).

### 3.2. Functional Enrichment Analysis of Shared DEGs

**Figure 2:**
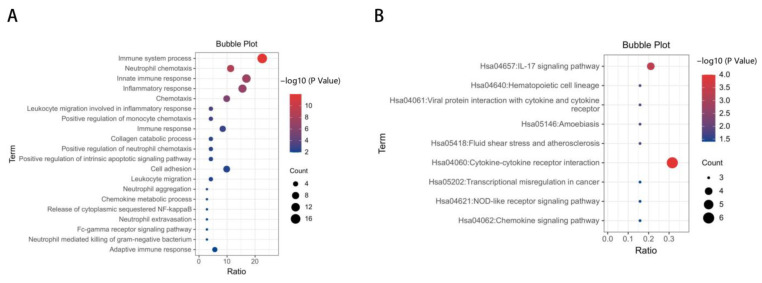
GO and KEGG enrichment analysis of DEGs. (**A**) Advanced bubble chart shows GO enrichment significance items of DEGs in biological processes. The *x*-axis label represents the gene ratio, and the *y*-axis label represents GO terms. (**B**) KEGG enrichment result of DEGs. The *x*-axis represents gene ratio and *y*-axis represents KEGG terms. The size of circle represents gene count. Different color of circles represents different adjusted *p* value.

To identify the biological processes associated with the 47 DEGs, GO annotation was accomplished by DAVID online tools. Most significant enrichment in BP of DEGs included inflammatory response, immune response, regulation of inflammatory response (Figure 2A, and Supplementary Table S2). In total, 19 genes enriched in inflammation-related biological processes in GO analysis were screened out and composed the “inflammation term”. To explore the enriched pathways of the inflammatory-related genes, KEGG pathway analysis was performed using DAVID online tools. Results of this analysis revealed that DEGs were mainly enriched in cytokine–cytokine receptor interaction, the IL-17 signaling pathway and hematopoietic cell lineage (Figure 2B, and Supplementary Table S3).

### 3.3. The Screening of Inflammation-Related Hub Genes and Involved Pathways via PPI Network Analysis

**Figure 3:**
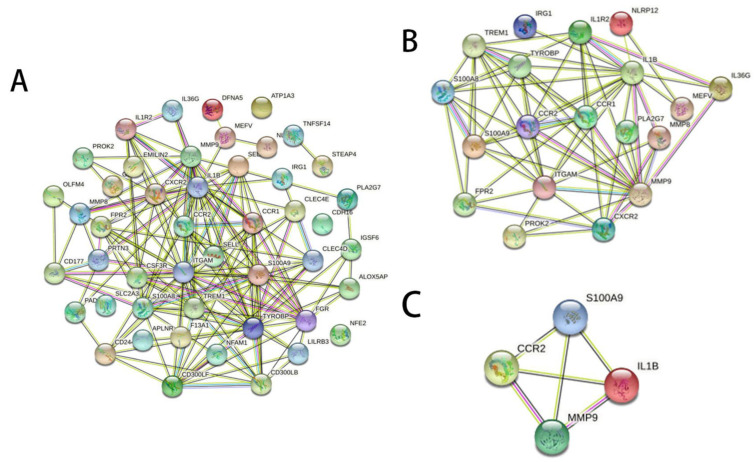
Gene interaction networks of DEGs. (**A**) PPI network of 47 DEGs which were homologous to the human counterparts. Nodes represent genes, edges represent interactions, and images inside each node represent the three-dimensional structure of each protein. (**B**) PPI network of the 19 inflammation-related DEGs. (**C**) PPI network of the four hub genes with the highest scores calculated by CytoHubba.

To calculate the inflammation-related hub genes and crucial gene modules involved in RILI progression, the online tool STRING (https://string-db.org/, assessed on 1 February 2022) and the Cytoscape software were used. Based on the STRING database, the 19 inflammation-related DEGs were filtered into the PPI network complex (Figure 3A,B and Supplementary Table S4), which contained 64 edges. Then, connectivity degree was calculated by a plug-in CytoHubba of Cytoscape software and four genes (fold changes ≥ 2) with the highest scores were considered as the hub genes. The results demonstrated that the four hub genes were S100A9, matrix metallopeptidase 9 (MMP9), IL-1β, and C-C motif chemokine receptor 1 (CCR1) (Figure 3C). Further KEGG analysis revealed that three hub genes (S100A9, MMP9, and IL-1β) were involved in the IL-17 signaling pathway (Table 2).

### 3.4. Construction of the RILI Mouse Model

**Figure 4:**
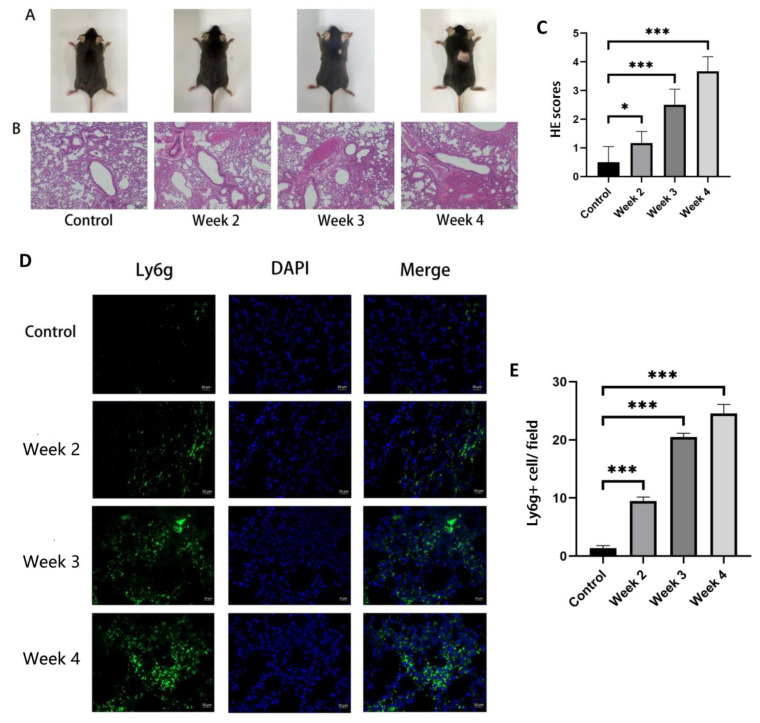
The change in fur appearance of the mice and the level of inflammatory cell infiltration in their lung tissue before and after irradiation. The mice in the control group did not receive irradiation, while the mice in the irradiation group were detected at 2, 3 and 4 weeks after irradiation. (**A**) After irradiation for 3–4 weeks, the back of mice showed hair loss. (**B**,**C**) H&E sections and inflammation scores of the lung tissues showed that inflammatory cell infiltration appeared in the lungs of irradiated mice with a time-dependent trend (bars, 200 μm). (**D**) Immunofluorescence of neutrophil Ly6g staining (green fluorescence) and DAPI nuclei staining (blue fluorescence) (bars, 20 μm). (**E**) Quantification of Ly6G (+) neutrophils per field of view. All graphs show the means ± SEM of at least three independent experiments. Statistical significance was analyzed by two-tailed *t*-test, * *p* < 0.05 and *** *p* < 0.001 compared to control.

Following the construction process outlined in “Materials and Methods”, obvious hair loss was observed in the irradiated mice at 3–4 weeks post irradiation, while there was no significant change in the mice at 1–2 weeks post irradiation (Figure 4A). By using H&E staining, the lung tissue from radiated mice was compared with that of the control group to identify the model of RILI. As described in the method, three randomly selected fields of each mouse were evaluated. As shown in the representative images in Figure 4B,C, compared with the control group, the group which received irradiation showed massive inflammatory cell infiltration in the alveoli. The degree of inflammatory cell infiltration was time dependent. The results of immunofluorescence staining of neutrophils showed that the number of neutrophils in the lung tissues increased with the time after irradiation. All of the above results showed the successful building of radiation-induced lung injury in the mouse model and the extent of injury was progressive with time.

### 3.5. Identification of S100A9 as a Potential Biomarker for RILI

**Figure 5:**
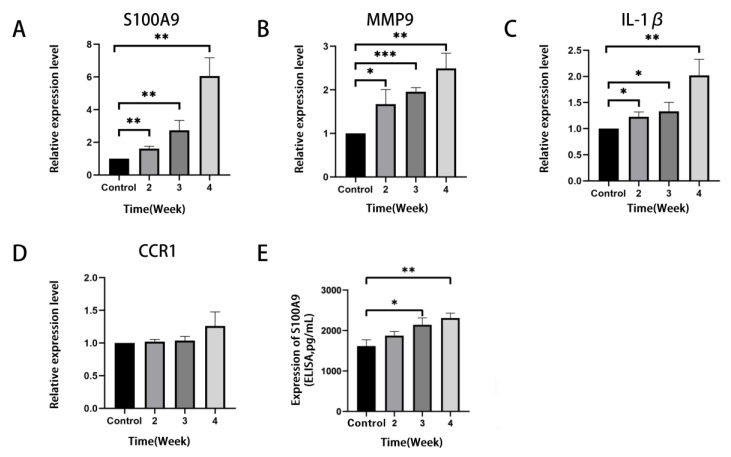
Verification of the expression level of the top four most significantly up-regulated DEGs in lung tissues of the mice before and after irradiation. The mice in the control group did not receive irradiation, while the mice in the irradiation group were detected at 2, 3 and 4 weeks after irradiation. (**A**–**D**) QRT-PCR validation of mRNA levels of S100A9, MMP9, IL-1β and CCR1 in mice lung tissues. The mRNA levels shown were the relative mRNA level compared to the mRNA level in the control group. (**E**) The level of S100A9 protein in BALF detected by ELISA. All graphs show the means ± SEM of at least three independent experiments. Statistical significance was analyzed by two-tailed *t*-test, * *p* < 0.05, ** *p* < 0.01, and *** *p* < 0.001 compared to control.

**Figure 6:**
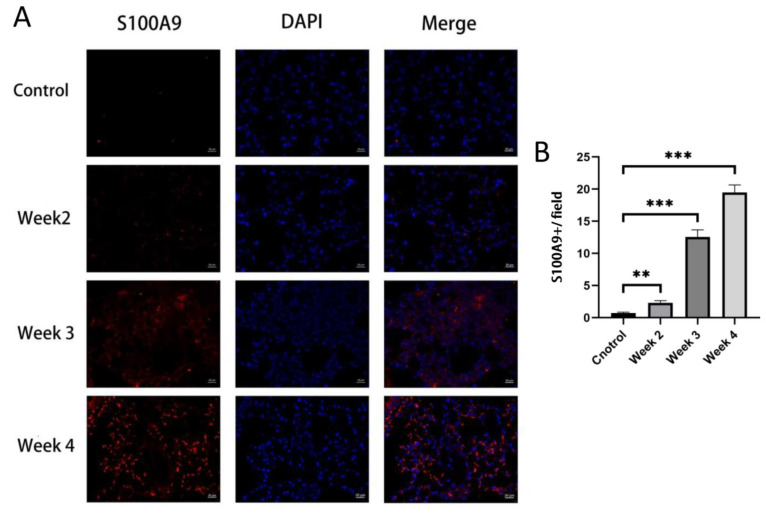
Immunofluorescence stained of S100A9 in lung tissues. The mice in the control group did not receive irradiation, while the mice in the irradiation group were detected at 2, 3 and 4 weeks after irradiation. (**A**) Immunofluorescence staining of S100A9 (red) and DAPI (blue) in lung tissues from normal and RILI mice (bars, 20 μm). (**B**) Quantification of the positive signals in immunofluorescence images of S100A9. All graphs show the means ± SEM of at least three independent experiments. Statistical significance was analyzed by two-tailed *t*-test, ** *p* < 0.01, and *** *p* < 0.001 compared to control.

To validate the results of the bioinformatics analysis in Section 3.3, the expression levels of the four hub genes in the lung of RILI mouse models and the controls were detected by qRT-PCR. The results showed that S100A9, MMP9 and IL-1β were significantly up-regulated in RILI mouse models with a time-dependent effects of radiation compared with controls (Figure 5A–D). The results were consistent with the analysis of microarray datasets. Furthermore, the expression of S100A9 showing the highest degree of overexpression among the four hub genes was measured in BALF by ELISA. It was demonstrated that the level of S100A9 in BALF in the RILI mouse model increased remarkably with the progression of inflammation (Figure 5E). For further study the role of S100A9 in RILI, we stained S100A9 in the lung tissues. The result of the immunostaining of lung sections revealed that the expression of S100A9 increased with the development of RILI (Figure 6A,B).

## 4. Discussion

RILI, a spectrum of toxicity to lung seen with thoracic radiotherapy for malignancy, is categorized into an acute injury stage, radiation pneumonitis (RP), and the chronic injury stage, radiation pulmonary fibrosis (RPF) [17]. The indications for radiation therapy are broadening with recent technological improvements, making the overall number of patients at risk of RILI significant. At present, systemic glucocorticoid treatment is taken as the main treatment of significantly symptomatic RP. Unfortunately, glucocorticoid also suffers from an array of side effects, including an increased risk of infection with pathogens [18]. There are no established guidelines for the treatment of RPF or even any effective therapies for it, and the treatment is primarily supportive. Preclinical studies demonstrate that some drugs may have efficacy in preventing lung fibrosis or reducing its progression rather than reversing it [19]. Therefore, it is particularly important to explore effective targets for early prediction and preventive intervention of RILI.

RILI involves a sequence of inflammatory events, which is characterized by the recruitment of diverse inflammatory cells and a perpetual cascade of cytokines and chemokines [9]. With the development of high-throughput sequencing technology, we can identify the specific inflammation-related biomarkers during the progression of RILI and find reliable targets for early diagnosis and prevention. In the present study, changes in the expression of critical genes were analyzed based on two GEO datasets (GSE25295 and GSE41789) during the development of RILI. A total of 75 DEGs were identified between RILI tissues and normal lung tissues, including 73 up-regulated genes and 2 down-regulated genes. GO analysis of BP demonstrated that DEGs were significantly enriched in the immune system process, innate immune response and inflammatory response. Studies have shown that radiation injury is related to immune disorder in tumor patients [20]. A great number of immune cells are involved in the occurrence and development of RILI, such as macrophages and lymphocytes. The infiltration of T lymphocytes to tumor and non-tumor microenvironments caused by radiotherapy is a major contributor to the initiation and extension of inflammation. Radiotherapy can also promote the immune response by strengthening antigen presentation in patients with abnormal immune function, so as to accelerate the development of radiation injury [21]. Th1/Th2 immune response is involved in RILI. When stimulated by external factors, primitive CD4+ T cells can be transformed into two cell subsets: Th1 cells and Th2 cells, secreting interferon-γ (IFN-γ), TNF-α, interleukin-2 (IL-2), interleukin-12 (IL-12) and interleukin-18 (IL-18) and inducing local inflammation [22]. After the exposure to radiation, alveolar type II epithelial cells produce a large number of pro-inflammatory cytokines, including IL-6 and TGF-β1 [23,24]. In addition, insulin-like growth factor-1 (IGF-1), platelet-derived growth factor-β (PDGF-β), Macrophage-derived chemokines (MDC) and other proinflammatory cytokines produced by macrophages and neutrophils can amplify inflammatory signals and aggravate local inflammation by enhancing the permeability of pulmonary capillary endothelium and the recruitment of monocytes, resulting in damage of alveolar structure [25]. As shown in the KEGG results, most of the genes we studied are enriched in the two signaling pathways of cytokine–cytokine receptor interaction and IL-17. Cytokine–cytokine receptor interaction is an important signaling pathway involved in a lot of biological process [26,27]. It is well known that cytokine–cytokine receptor interaction can participate in inflammatory response [28], which could further promote the development of RILI. The IL-17 signaling pathway is another significant signaling pathway [29]. IL-17 is a proinflammatory cytokine, which is produced mostly by T-helper 17 cells [30]. Relevant studies have proved that IL-17 can play a role in pneumonia and pulmonary fibrosis by mediating inflammatory response [31]. More interestingly, IL-17 can also change the secretion of S100A9 [32]. Therefore, we speculate that IL-17 can change the occurrence and development of RILI by mediating the secretion of S100A9.

In this study, four genes were finally screened out as early inflammation-related biomarkers of RILI—MMP9, IL-1β, CCR1 and S100A9. MMP9 is a kind of matrix metalloproteinases which can degrade extracellular matrix and a lot of non-matrix proteins. It promotes the development of pulmonary fibrosis by promoting abnormal epithelial cell migration and other abnormal repair processes [33]. IL-1β is a family member of interleukin-1 (IL-1), possessing pro-inflammatory effects [34] and associated with pulmonary interstitial fibrosis [35]. CCR1 is a cytokines receptor and essential in the recruitment of interstitial leukocyte, inducing inflammatory response [36]. S100A9, belonging to the S100 family, plays an important role in trauma, infection, high temperature, stress and many other inflammatory processes [37]. S100A9 is mainly stored in immune cells such as macrophages and neutrophils, and is rapidly released under inflammatory stimulation, and then plays a role as a damage-related molecular model (DAMP) [38]. Our study revealed that S100A9, MMP9 and IL-1β were time dependently up-regulated in RILI, and this was verified in animal models.

In conclusion, our study identified four hub genes via microarray and bioinformatics analysis, among which the expression level of S100A9 was increased with the development of RILI and inflammation. We provide a potential target for early diagnosis of the development of RILI. These results may be helpful for finding the specific mechanism in RILI.

## Figures and Tables

**Table 1:** Primers for quantitative polymerase chain reaction.

Gene Name	Forward	Reverse	Product Length, bp
Gapdh	TGTGTCCGTCGTGGATCTGA	CCTGCTTCACCACCTTCTTGA	77
MMP9	GGACCCGAAGCGGACATTG	CGTCGTCGAAATGGGCATCT	139
S100A9	ATACTCTAGGAAGGAAGGACACC	TCCATGATGTCATTTATGAGGGC	129
IL-1B	GAAATGCCACCTTTTGACAGTG	TGGATGCTCTCATCAGGACAG	116
CCR1	ATACTCTGGAAACACAGACTCACT	TCCTTTGCTGAGGAACTGGTC	84

**Table 2:** Results of KEGG signaling pathway analyses.

KEGG Pathway	Description	Count	*p* Value
hsa04657	IL-17 signaling pathway	3	1.5 × 10^−4^
hsa04668	TNF signaling pathway	2	3.36 × 10^−2^
hsa05418	Fluid shear stress and atherosclerosis	2	3.36 × 10^−2^

## Data Availability

The data presented in this study are openly available in the GEO at https://www.ncbi.nlm.nih.gov/geo/query/acc.cgi?acc=GSE25295, assessed on 10 January 2022; https://www.ncbi.nlm.nih.gov/geo/query/acc.cgi?acc=GSE41789, assessed on 10 January 2022.

## Supplementary Materials

The following supporting information can be downloaded at: https://www.mdpi.com/article/10.3390/jcm12030733/s1. Table S1: Differentially expressed genes of RILI; Table S2: Results of GO functional annotation analyses; Table S3: Results of KEGG signaling pathway analyses; Table S4: Differentially expressed genes related to inflammation.
