# Supplementary material for: Identification of S100A9 as a Potential Inflammation-Related Biomarker for Radiation-Induced Lung Injury
Source: J Clin Med. 2023 Jan 17;12(3):733. doi: 10.3390/jcm12030733 (PMC9917937; doi:10.3390/jcm12030733)
Supplement: Supplementary file 1 [file jcm-12-00733-s001.zip › Table S1.pdf]

Table S1. Differentially expressed genes of RILI

DEGs	Genes name
Up-regulated	Clec4e Wfdc17 Csf3r Trem1 Pla2g7 Il1f9 Cxcr2 Slfn4 S100a9 Ngp S100a8 Mmp8 Il1r2 Stfa2l1 Slc2a3 Mmp9 Slfn4 Mrgpra2a Wfdc21 Clec4d Pirb Ccr1 Il1b Fgr Pirb 9530053H05Rik Stfa1 Dfna5 Itgam LOC432459 Tnfsf14 Cd300lf Slfn1 Gda Emilin2 Mefv Ifitm6 LOC677008 Nfe2 Tlr13 Steap4 Sell Selplg Cyp4f18 Sirpb1a Cd300lb Sirpb1b Alox5ap AB124611 Ceacam10 Padi4 Nfam1 Cd177 Igsf6 Lilrb4a F13a1 Clca3a2 Gm14548 Fpr2 Acod1 Clca3a1 Olfm4 Trem3 Prtn3 Nlrp12 Cd244 Tyrobp Atp1a3 Prok2 H2-Q10 Ccr2 Saa3
Down-regulated	Cdh16 Aplnr
