# Supplementary material for: Identification of S100A9 as a Potential Inflammation-Related Biomarker for Radiation-Induced Lung Injury
Source: J Clin Med. 2023 Jan 17;12(3):733. doi: 10.3390/jcm12030733 (PMC9917937; doi:10.3390/jcm12030733)
Supplement: Supplementary file 1 [file jcm-12-00733-s001.zip › Table S2.pdf]

Table S2. Results of GO functional annotation analyses

GO term	Description	Count	*P* Value
GO:0002376	immune system process	16	1.01×10^{-12}
GO:0030593	neutrophil chemotaxis	8	2.51×10^{-9}
GO:0045087	innate immune response	12	5.84×10^{-8}
GO:0006954	inflammatory response	11	1.50×10^{-7}
GO:0006935	chemotaxis	7	2.31×10^{-6}
GO:0002523	leukocyte migration involved in inflammatory response	3	7.98×10^{-4}
GO:0090026	positive regulation of monocyte chemotaxis	3	9.29×10^{-4}
GO:0006955	immune response	6	1.92×10^{-3}
GO:0030574	collagen catabolic process	3	2.76×10^{-3}
GO:0090023	positive regulation of neutrophil chemotaxis	3	3.75×10^{-3}
GO:2001244	positive regulation of intrinsic apoptotic signaling pathway	3	4.29×10^{-3}
GO:0007155	cell adhesion	7	4.89×10^{-3}
GO:0050900	leukocyte migration	3	5.49×10^{-3}
GO:0070488	neutrophil aggregation	2	6.52×10^{-3}
GO:0050755	chemokine metabolic process	2	6.52×10^{-3}
GO:0008588	release of cytoplasmic sequestered NF-kappaB	2	9.76×10^{-3}
GO:0072672	neutrophil extravasation	2	9.76×10^{-3}
GO:0038094	Fc-gamma receptor signaling pathway	2	9.76×10^{-3}
GO:0070945	neutrophil mediated killing of gram-negative bacterium	2	9.76×10^{-3}
GO:0002250	adaptive immune response	4	1.06×10^{-2}
GO:0019221	cytokine-mediated signaling pathway	4	1.21×10^{-2}
GO:0007204	positive regulation of cytosolic calcium ion concentration	4	1.25×10^{-2}
GO:0002793	positive regulation of peptide secretion	2	1.30×10^{-2}
GO:0042107	cytokine metabolic process	2	1.30×10^{-2}
GO:0002374	cytokine secretion involved in immune response	2	1.30×10^{-2}
GO:0002292	T cell differentiation involved in immune response	2	1.30×10^{-2}
GO:0018119	peptidyl-cysteine S-nitrosylation	2	1.62×10^{-2}
GO:0050729	positive regulation of inflammatory response	3	1.80×10^{-2}
GO:1900016	negative regulation of cytokine production involved in inflammatory response	2	2.26×10^{-2}
GO:0032496	response to lipopolysaccharide	4	2.65×10^{-2}
GO:0050728	negative regulation of inflammatory response	3	3.28×10^{-2}
GO:0007229	integrin-mediated signaling pathway	3	3.70×10^{-2}
GO:0045824	negative regulation of innate immune response	2	4.79×10^{-2}
GO:0030502	negative regulation of bone mineralization	2	4.79×10^{-2}
GO:0010820	positive regulation of T cell chemotaxis	2	4.79×10^{-2}
GO:0014002	astrocyte development	2	5.41×10^{-2}

GO:0043122	regulation of I-kappaB kinase/NF-kappaB signaling	2	5.41×10^{-2}
GO:0006968	cellular defense response	2	5.41×10^{-2}
GO:0045766	positive regulation of angiogenesis	3	5.94×10^{-2}
GO:0033198~	response to ATP	2	6.33×10^{-2}
GO:0030595	leukocyte chemotaxis	2	6.94×10^{-2}
GO:0035987	endodermal cell differentiation	2	8.45×10^{-2}
GO:0001819	positive regulation of cytokine production	2	9.05×10^{-2}
