# Supplementary material for: Identification of S100A9 as a Potential Inflammation-Related Biomarker for Radiation-Induced Lung Injury
Source: J Clin Med. 2023 Jan 17;12(3):733. doi: 10.3390/jcm12030733 (PMC9917937; doi:10.3390/jcm12030733)
Supplement: Supplementary file 1 [file jcm-12-00733-s001.zip › Table S3.pdf]

Table S3. Results of KEGG signaling pathway analyses

KEGG pathway	Description	Count	*P* Value
hsa04657	IL-17 signaling pathway	4	4.5×10^{-4}
hsa04640	Hematopoietic cell lineage	3	1.49×10^{-2}
hsa04061	Viral protein interaction with cytokine and cytokine receptor	3	1.49×10^{-2}
hsa05146	Amoebiasis	3	1.49×10^{-2}
hsa05418	Fluid shear stress and atherosclerosis	3	2.07×10^{-2}
hsa04060	Cytokine-cytokine receptor interaction	6	1×10^{-4}
hsa05202	Transcriptional misregulation in cancer	3	3.9×10^{-2}
hsa04621	NOD-like receptor signaling pathway	3	3.9×10^{-2}
hsa04062	Chemokine signaling pathway	3	3.9×10^{-2}
