# Supplementary material for: Identification of S100A9 as a Potential Inflammation-Related Biomarker for Radiation-Induced Lung Injury
Source: J Clin Med. 2023 Jan 17;12(3):733. doi: 10.3390/jcm12030733 (PMC9917937; doi:10.3390/jcm12030733)
Supplement: Supplementary file 1 [file jcm-12-00733-s001.zip › Table S4.pdf]

Table S4. Differentially expressed genes related to inflammation

DEGs	TREM1 IRG1 IL1R2 NLRP12 S100A8 TYROBP IL1B S100A9 CCR2 CCR1 FPR2 ITGAM PROK2 CXCR2 MMP9 PLA2G7 MMP8 MEFV IL36G
